# The Protective Effects of Different-Time-Ischemic Preconditioning on the Reperfusion Injury in Fatty Livers in Rats

**DOI:** 10.1371/journal.pone.0058086

**Published:** 2013-03-06

**Authors:** Yong Jiang, Jian Jun Tang, Bao Qiang Wu, Bo Yuan, Zhen Qu

**Affiliations:** 1 Department of Hepatobiliary Surgery, the 1st people’s hospital of Changzhou, Changzhou, Jiangsu Province, People’s Republic of China; 2 Department of Hepatobiliary Surgery, People’s Hospital of Wujin, Changzhou, Jiangsu Province, People’s Republic of China; University College London, United Kingdom

## Abstract

**Background:**

The present study was aimed to investigate the protective effects of different-time-ischemic preconditioning on the reperfusion injury in fatty livers in rats, and to elucidate the mechanisms underlying the protective effects and the optimal safe ischemic preconditioning time on the hepatic IR injury in steatotic livers.

**Methodology/Principal Findings:**

A rat fatty liver model was established by high-fat diet feeding. We investigated the changes in the concentration of AST, ALT, LDH and NO in the serum, and of MDA, SOD, and MPO in the liver samples in response to different ischemic preconditioning times and ischemia-reperfusion injury. Histological analysis was performed to evaluate the results of the hepatic fatty infiltration. 1) At 24 h after 15 min ischemic preconditioning with 10 min reperfusion (15 min +10 min IP), the extent and area of the necrosis was markedly higher in the fatty liver samples with respect to IR, compared to the normal liver samples. 2) In response to the treatment of 5/8 min +10 min IP, the fatty liver group showed lower levels of serological indicators and liver MDA and MPO compared to the other groups, while the SOD activity of the fatty liver group was significantly higher than the other groups (p<0.05). Compared to the corresponding IR group, all IP groups showed a significantly higher serum NO concentration (p<0.05). Among the fatty liver groups, the 5/8 min+10 min IP group showed the highest NO concentration (p<0.05).

**Conclusions/Significance:**

Fat infiltration could aggravate the ischemia-reperfusion injury in the rat liver. Furthermore, ischemic preconditioning could increase the tolerance of the fatty liver, which was induced by the high-fat diet, to hepatic ischemia-reperfusion injury in rats. The protocol of 5/8 min +10 min IP was the optimal regimen for the treatment of moderate and severe fatty livers.

## Introduction

Hepatic ischemia-reperfusion injury is an important reason for post-surgical liver dysfunction, especially for liver resection and liver transplantation. Hepatic steatosis is a major risk factor for liver damage, because the fatty liver can reduce the tolerance of the liver to ischemia-reperfusion injury. It has been suggested that hepatectomy at room temperature to treat fatty liver ischemia can result in liver failure. Furthermore, liver transplantation using a fatty donor liver has a higher risk of post-surgical primary non-function and dysfunction [1].

In the present study, we established a non-alcoholic rat fatty liver model by means of high-fat diet feeding. Using this model, we investigated the changes in the concentrations of serum enzymes (i.e. aspartic transaminase (AST), alanine aminotransferase (ALT), lactic dehydrogenase (LDH), and nitric oxide (NO)) and hepatic cytokines (i.e. malondialdehyde (MDA), superoxide dismutase (SOD), and myeloperoxidase (MPO)) in response to different ischemic preconditioning times and ischemia-reperfusion injury, to explore the optimal time of ischemic preconditioning for the treatment of moderate and severe fatty livers, and the underlying mechanisms.

## Materials and Methods

The animal experiments were approved by the Animal Care and Use Committee of the Third Affiliated Hospital of Suzhou University, Changzhou, Jiangsu, P.R.China.

### Animal

126 male SD rats of clean grade (weight 140–160 g) were randomly divided into 7 groups (Table 1). The test groups (C-G) were fed a high-fat diet, which was composed of 2% cholesterol, l2% lard, and 86% normal diet [2]. The control groups (A and B) were fed a normal diet. All animals were fed for three weeks. The animal room was well ventilated with a room temperature of 20–22°C, and a day/night cycle of 12 h.

**Table 1:** Animal groups and treatments.

Group	Diet	Preconditioning		Title
		Ischemia	Reperfusion	
A	Normal	Non-preconditioning		IR
B		10 min	10 min	IP-10
C	High-fat	Non-preconditioning		IR
D		10 min	10 min	IP-10
E		15 min	10 min	IP-15
F		5 min	10 min	IP-5
G		8 min	10 min	IP-8

**Figure 1:**
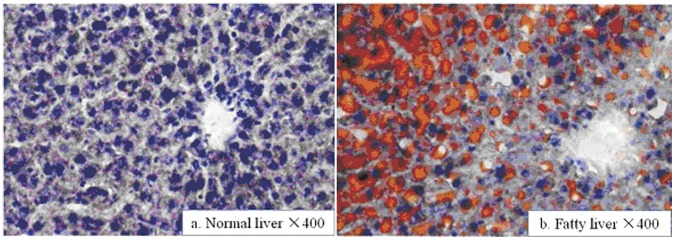
Difference of steatosis in the livers of fatty vs. normal Sprague-Dawley(SD) rats using hematoxylin-eosin(HE) staining. (a) Normal SD rats show no evidence of steatosis. (b) Fatty SD rats show fatty infiltration in hepatocytes. Hematoxylin-eosin(HE) staining; original magnification ×400.

### Surgical Procedure and Sample Collection

To establish the ischemia-reperfusion model, the animals were given ischemic preconditioning (Table 1), followed by an ischemia-reperfusion injury procedure (portal triad clamping for 30 min, and blood flow restoration for 30 min). In each group, blood samples were collected from the inferior vena cava of 6 rats at 1, 4, and 24 h after blood flow restoration. Serum was isolated through centrifugation at 4000 r/min at 4°C for 3 min, and stored at −80°C for use. 24 h later, liver samples were collected and stored either in liquid nitrogen for future use, or in formaldehyde for HE staining.

**Figure 2:**
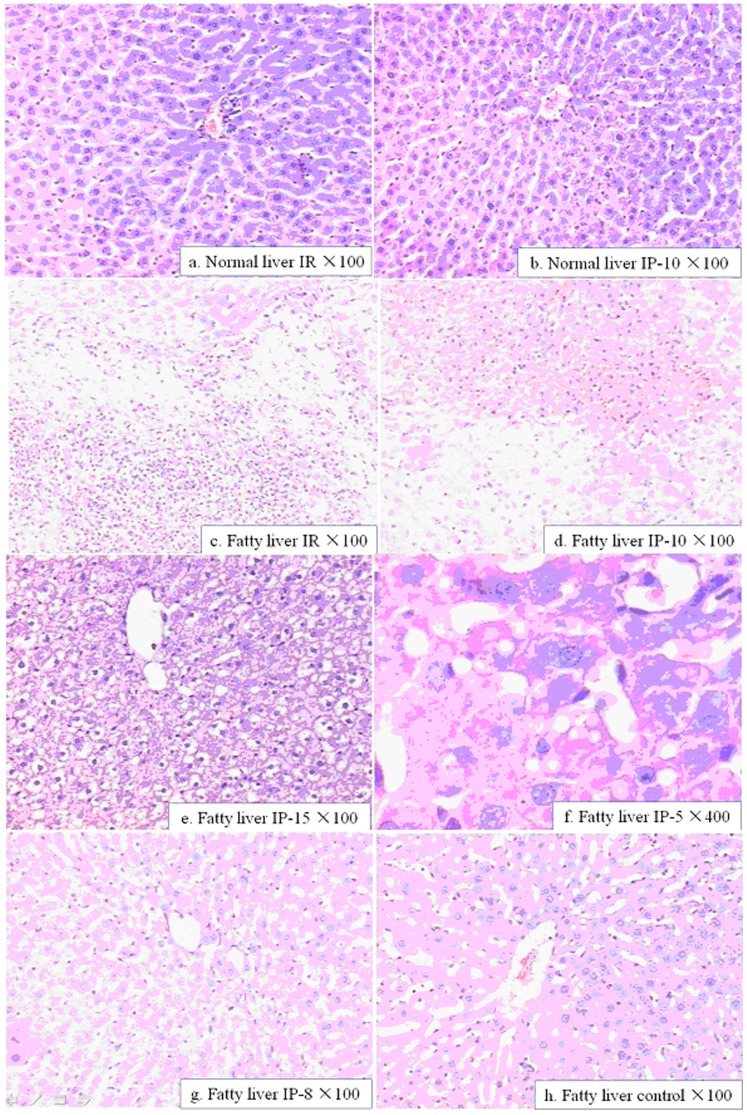
Histological lesions in liver at 24 hours of hepatic reperfusion. (a) I/R(normal): small area of hepatocyte necrosis and coagulative necrosis with neutrophil infiltration. (b) IP-10(normal): slight incipient and coagulative patchy necrosis of isolated hepatocytes. (c) I/R(fatty): extensive and confluent areas of coagulative necrosis, neutrophilic infiltration, and hemorrhage were observed. (d) IP-10(fatty): patchy areas of hepatocyte incipient necrosis and scattered multifocal areas of coagulative necrosis with neutrophil infiltration were observed, similar to (e) IP-15 (fatty). (f) IP-5(fatty) (original magnification ×400): irregular area of hepatocyte incipient necrosis with neutrophil infiltration, similar to (g) IP-8(fatty), the percentage of necrosis was significantly less in preconditioned than in unpreconditioned group. (h) fatty liver: severe fatty infiltration in hepatocytes as contrl. Hematoxylin-eosin(HE) staining; original magnification ×100.

### Examination

1. Histological examination using the HE staining method was performed to investigate the morphological changes in the liver cells in response to different reperfusion times.
2. Examination of the concentrations of liver damage indicators in the serum: Serum samples were analyzed using the automatic biochemical analyzer to assay the enzyme concentrations at different reperfusion time points. The enzymes included AST, ALT, and LDH. The concentration of NO in the serum was measured using the nitrate reduction method. Serum was separated by centrifugation and stored at −80°C before use. Nitrite was measured after enzymatic conversion by nitrate reductase using the Griess reaction, as described by Schmidt. Values obtained represented the sum of serum nitrite and nitrate.
3. Examination of makers of oxidation and neutrophil activation in liver: Liver samples stored in liquid nitrogen were thawed in ice water. 10 g liver sample was used to prepare the 10% liver tissue homogenate. Lipid peroxidation, used as an indirect index of the oxidative injury induced by the reactive oxygen species, was determined by measuring the formation of MDA in the liver with the thiobarbiturate reaction. MPO was used as a marker of hepatic neutrophil infiltration. MPO activity was measured photometrically employing 3, 3?, 5, 5′-tetramethylbenzidine as a substrate. Frozen liver tissues were macerated, homogenized, sonicated, and centrifuged at 4,000 g for 12 minutes at 4°C as described previously. MPO activity was measured in the supernatant, with calculations based on the absorbance change at 460 nm. All values were normalized to tissue weight. The amount of SOD activity from hepatic homogenates was measured with a commercial SOD assay kit.

### Statistical Analysis

All data were shown as mean value ± standard deviation. Statistical analysis was performed using ANOVA. A significant difference was determined as p<0.05.

**Figure 3:**
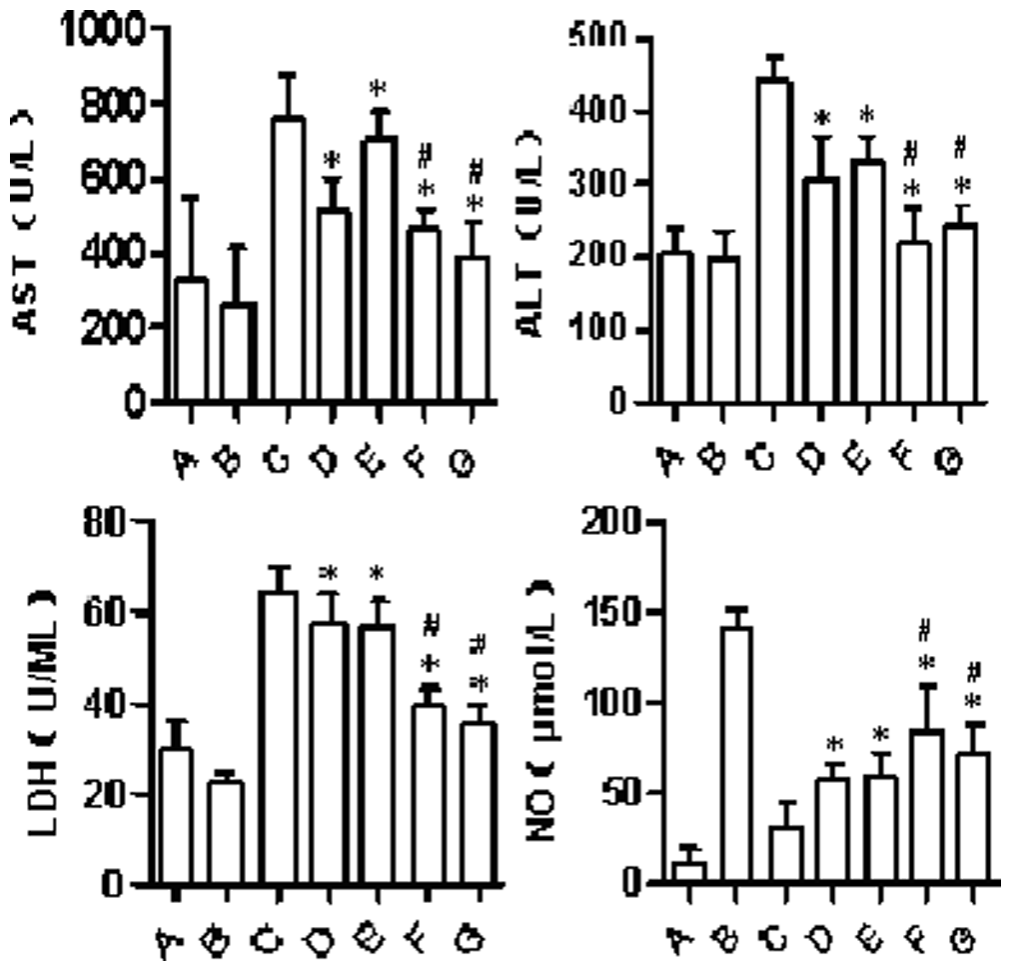
Concentration of ALT, AST, LDH, and NO at 4 h after the ischemia-reperfusion injury. *P<0.05, compared to the group C; # P<0.05, compared to the groups D and E.

## Results

### Animal Profile

The control group rats had glossy coats and good appetite, and were active. The fatty liver group rats ate more, and were sedentary and sleepy. At the end of the third week, the weight was 255.08±30.31 g for the control group and 261.17±27.26 g for the fatty liver group, and the difference was not significant.

**Table 2:** Concentration of ALT, AST, LDH, and NO at 4 h after the ischemia-reperfusion injury (x±s).

Indicator	Control		Fatty liver				
	A	B	C	D	E	F	G
AST (U/L)	326.5±227.4	262.3±149.9	759.3±120.6	514.8±88.2*	706.4±72.4*	460.3±54.5* #	391.4±89.3* #
ALT (U/L)	205.7±32.6	196.1±38.5	440.9±30.7	308.2±55.9*	328.7±34.7*	219.5±46.9* #	243.5±28.8* #
LDH (U/ML)	29.5±6.4	22.3±2.4	64.3±5.6	57.9±5.9*	57.0±5.5*	39.6±3.4* #	35.1±4.5* #
NO (µmol/L)	11.4±8.5	141.5±10.3	31.7±13.3	57.4±9.2*	59.0±13.6*	84.2±25.5* #	71.8±16.6* #

* P<0.05, compared to the control group;

# P<0.05, compared to the groups D and E.

### Pathological Changes in the Liver

Changes in the lipid content and inflammatory infiltration of the liver were assessed by two pathologists blinded to the animal groups in order to ensure objectivity in the histological assessments.

**Figure 4:**
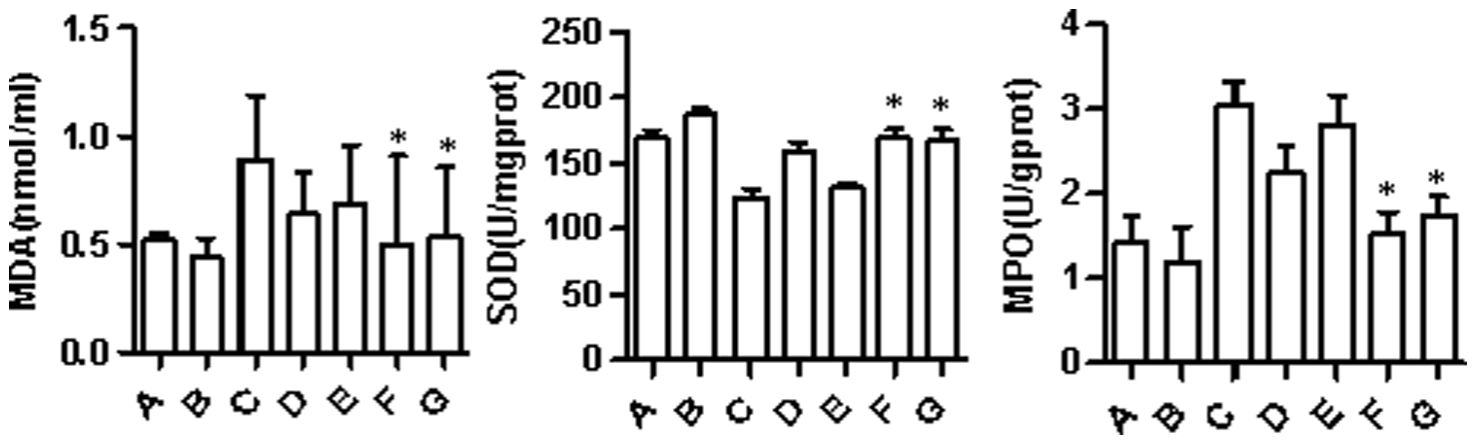
Changes in the concentration of MDA, SOD, and MPO in response to the ischemia-reperfusion injury. *P<0.05, compared to the groups C, D, and E.

**Table 3:** Changes in the concentration of MDA, SOD, and MPO in response to the ischemia-reperfusion injury (x±s).

Indicators	Control		Fatty liver				
	A	B	C	D	E	F	G
MDA(nmol/ml)	0.52±0.03	0.44±0.09	0.89±0.30	0.64±0.19	0.69±0.27	0.50±0.41*	0.54±0.32*
SOD(U/mgprot)	169.54±5.60	186.77±4.41	123.82±6.42	158.44±7.58	131.72±2.68	169.04±6.35*	167.19±8.98*
MPO(U/gprot)	1.42±0.32	1.20±0.42	3.05±0.28	2.26±0.31	2.82±0.35	1.53±0.26*	1.75±0.23*

* P<0.05, compared to the groups C, D, and E.

#### 1 Changes in the lipid content of the liver (Figure 1)

Fatty liver was determined as that more than 30% of the liver cells showed steatosis in a low magnification view [3]. All rats fed a high-fat diet had either moderate (30%–60%) or severe (>60%) fatty livers, thus the fatty liver model was successfully established. None of the rats in the control group showed hepatic steatosis.

#### 2 Inflammatory infiltration of the liver (Figure 2)

In the high magnification view, the fatty liver IR group showed a large degree of infiltration of neutrophils and lymphocytes. Among the fatty liver IP groups, the 15 min +10 min IP group showed the greatest degree of pathological changes and inflammatory infiltration, which, however, was still lower than the reperfusion group (P<0.05). These results indicated that a short-term blood supply termination and restoration before ischemia-reperfusion had a protective effect on the liver function. Furthermore, among the fatty liver IP groups (D–G), groups F (5 min +10 min IP) and G (8 min +10 min IP) showed the lowest degree of infiltration (P<0.05), indicating that the protocol of 5/8 min +10 min IP was probably the optimal regimen for the treatment of ischemia-reperfusion injury. In contrast, the control group rats showed moderate inflammatory infiltration in the hepatic lobule and portal area compared to the fatty liver groups.

### Changes in the Concentrations of Serum ALT, AST, LDH, and NO (Table 2; Figure 3)

At 4 h after reperfusion, the concentrations of ALT, AST and LDH reached the highest level for all groups, indicating the highest degree of liver injury at this time point (P<0.05). Furthermore, the ischemic preconditioning groups showed lower concentrations of ALT, AST and LDH, compared to other groups.

Importantly, the ischemic preconditioning groups showed a significantly higher concentration of NO compared to corresponding reperfusion injury groups (C and D) (P<0.05). Among the ischemic preconditioning groups, groups F and G showed obviously higher NO concentrations than groups D and E (P<0.05), indicating that a 5 or 8 min ischemia can maximally protect the liver. In addition, among the preconditioning groups, the serum NO concentration reached the highest level at 4 h compared to 1 h and 24 h (P<0.05).

### Changes in the Concentrations of MDA, SOD, and MPO in the Liver (Table 3; Figure 4)

Examination of the concentrations of MDA, SOD, and MPO in the liver homogenate: Fatty liver group C showed a significantly higher MDA concentration than groups D–G (P<0.01), while group F showed the lowest concentration. Group F showed a significantly higher SOD concentration than groups C–E (P<0.05), which, however, was only slightly higher than that of group G. The MPO concentration was the highest in group C and the lowest in group F (P<0.05). Control groups A and B showed significant differences in all comparisons, including SOD, MDA, and MPO (P<0.05). Comparing the fatty liver groups and the corresponding control group, significant differences were observed between groups A and C, and between groups B and D (P<0.05).

## Discussion

### Hepatic Steatosis Resulted in the Deterioration of the IR Injury

Compared to the normal liver, the fatty liver is more vulnerable to ischemia-reperfusion injury. Our biochemical and pathological analyses revealed that the fatty liver had a poor tolerance to 30 min hepatic ischemia. MDA is the product of free radical lipid peroxidation [4]. Compared to the normal liver, IR can induce a more severe injury to the fatty liver, and subsequently cause changes in a variety of cytokines. Thus, ischemic preconditioning which consisted of a short 5 or 8 min ischemia and a 10 min reperfusion, showed a strong protective effect against the following 30 min ischemia-reperfusion injury, and subsequently increased the survival rate of rats with fatty livers in response to 30 min ischemia.

### The Protective Effects of Ischemic Preconditioning on the Reperfusion Injury in Fatty Livers in Rats

For fatty livers, the liver cells are supposed to be more vulnerable to lipid peroxidation and excessive reactive oxygen. SOD plays a vital role in the maintenance of the oxidative antioxidant balance [5], and its activity represents the capability of the body to scavenge free radicals. In the present study, ischemic preconditioning can attenuate the lipid peroxidation reaction in the fatty liver after reperfusion. As the animals were randomly grouped, the precondition groups and the non-preconditioning groups should have similar degrees of liver steatosis. Hence, the attenuation of the lipid peroxidation by ischemic preconditioning was probably caused by the ischemia induced reduction in the production of reactive oxygen.

MPO is enriched in neutrophils, and the activity of MPO can be used to estimate the number of neutrophils [6]. In the present study, the results about the changes in the hepatic MPO and MDA concentrations indicated that the preconditioning attenuated the IR induced aggregation of neutrophils in the liver. Furthermore, pathological analysis also indicated that IP significantly reduced the inflammatory infiltration in the liver.

The multiple mechanisms underlying the IR induced injury in both the normal and fatty livers indicated that drugs might not be effective in the treatment of IR induced liver injury [7]. Hence, ischemic preconditioning can be used as a new, important clinical approach. Our results indicated that the mechanisms of the protection effects of ischemic preconditioning on the IR induced fatty liver injury could be through the NO pathway mediated attenuation of lipid peroxidation, the reduction of neutrophil accumulation, and improvement in the liver’s microcirculation.

NO is considered the best candidate to mediate the protection effects of preconditioning. A previous study has confirmed the protection effects of ischemic preconditioning on the reperfusion injury in the liver [8]. Previous studies of NO synthase inhibitors using different liver IR models suggested that NO may take protection effects by improving the hepatic microcirculation, and reducing the neutrophil accumulation and oxidative stress.

### The Differential Effects of Different-time-ischemic Preconditioning on the Reperfusion Injury in Fatty Livers in Rats

Our study indicated that ischemic preconditioning can enhance the tolerance of rat fatty livers to reperfusion injury. All our results of liver enzyme concentration changes, pathological alterations, and inflammatory infiltration indicated that there were significant differences between the fatty liver IR groups and the IP groups. Importantly, the varied preconditioning time showed either slightly or significantly different protection effects. Particularly, the protocol of 5 or 8 min ischemic preconditioning with 10 min reperfusion had the strongest protection effects on the moderate and severe fatty livers, whereas the protocol of 15 min preconditioning with 10 min reperfusion induced pathological and enzymatic changes analogous to the IR groups. Moreover, with the increased preconditioning time, the liver injury became more severe, with such as structural disorder of the liver, cell swelling, sinus congestion, inflammation, and large homogeneous structure of the red dye necrosis.

In summary, our study suggested that ischemic preconditioning can attenuate reperfusion injury in the liver through an NO involved mechanism. The protocol of 5 or 8 min ischemic preconditioning with 10 min reperfusion had the strongest protection effects on moderate and severe fatty livers. With the increase in the degree of hepatic steatosis, less preconditioning time was needed for the protection effects to take place.
